# A novel and innovative device to retract rectum during radiation therapy of pelvic tumors

**DOI:** 10.1002/acm2.12517

**Published:** 2018-12-26

**Authors:** E. Ishmael Parsai, Ahmadreza Jahadakbar, Hossein Lavvafi, Mohammad Elahinia

**Affiliations:** ^1^ Department of Radiation Oncology University of Toledo Medical Center Toledo OH USA; ^2^ Department of Mechanical Industrial and Manufacturing Engineering University of Toledo Toledo OH USA

**Keywords:** brachytherapy, external beam radiation therapy, prostate Cancer, pelvic tumors, rectal retractor

## Abstract

An effective radiotherapy treatment entails maximizing radiation dose to the tumor while sparing the surrounding and normal tissues. With the advent of SBRT with extreme hypo‐fractionation in treating tumors including prostate where ablative dose is delivered in smaller number of fractions, rectum remains a dose‐limiting organ and at the risk of rectal toxicity or secondary cancer. The same limitation of rectal toxicity exists for high‐dose rate (HDR) treatments of cervical, endometrial, or prostate cancer when creating even a short distance between the anterior rectal wall and field of radiation is ideal in delivering ablative dose to the target. An effective solution to such problem is to physically displace rectum as the organ at risk. This research presents an organ retractor device that is designed to displace the rectum away from the path of radiation beam employing a Nitinol shape memory alloy that is designed for displacing the rectum upon actuation. A control system regulates the motion in a reproducible and safe manner by creating the desirable shape in moving the anterior rectal wall. The study finds the novel organ retractor device to be a promising tool that can be applied in a clinical setting for minimizing dose to the rectum during treatment of pelvic tumors, and creating the potential to deliver an ablative dose to tumor volume or to escalate the dose when needed.

## 1 INTRODUCTION

**Figure 1:**
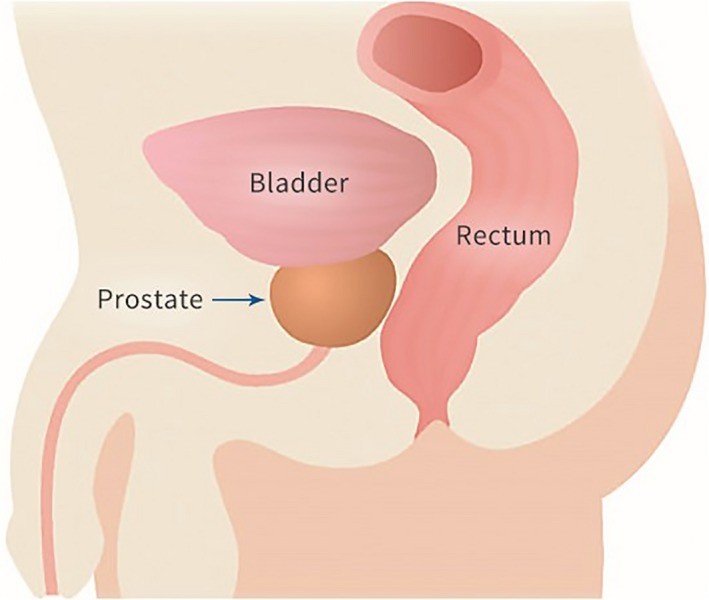
Anatomical positions of rectum and prostate.

Due to the proximity of the prostate to the anterior wall of the rectum, some portions of the rectal wall are often exposed to high doses of ionizing radiation whether in external beam radiotherapy or in brachytherapy treatments as seen in Fig. 1.

For example, due to the radiation exposure to neighboring critical structures during prostate cancer treatment, a significant increase in cancer risk was observed for the bladder (77%) and the rectum (105%) over the following decade.^1^ Although uncommon, available data show some patients develop transfusion‐dependent rectal bleeding, ulcers, or fistulas.^2^ Rectal toxicity complications may require permanent colostomy and thus can significantly impact patients’ quality of life (QOL).^2^, ^3^ Moving the rectum away even a short distance from the plane of radiation can relax the prescription dose limitation.

Some strategies have been recently adopted and implemented that can potentially help minimizing these complications.^4^, ^5^, ^6^ For instance, by injecting biodegradable materials behind Denonviller's fascia, one can increase the distance between the rectum and the radiation/radioactive sources to consequently decrease the rectal dose and minimizing the side effects.^4^, ^5^ This paper summarizes the progress in this area, issues with current strategies and finally presents a novel approach with significantly lower cost, minimally invasive procedure, and minimal to no complications.

**Figure 2:**
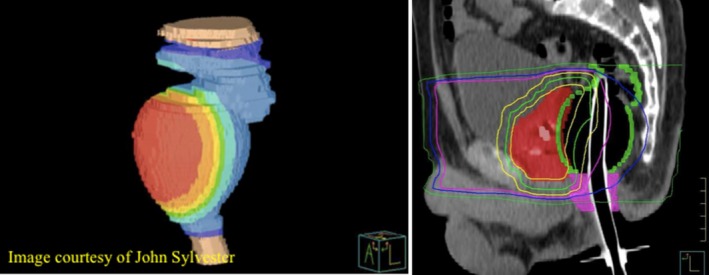
Radiation dose received by the rectal wall when using rectal balloon (John Sylvester). [Correction added on 3 January 2019, after first online publication: the article has been corrected for Figure 2 after original publication.]

There are several methods that have been suggested and used clinically to spare rectum for patients undergoing prostate radiation treatment. For instance, one technique is used to fixate the prostate gland during the course of radiation treatment via a rectal balloon to reduce the prostate motion and to make sure the dose delivered to the target volume is efficient.^6^ Prostate immobilization utilizing the rectal balloon permits a safer and smaller planning target volume margin as stated elsewhere.^7^ While such technique allows for minimizing the anteroposterior and lateral prostate displacements and reduces the dose to the posterior region of the rectum, the anterior part of the rectum will receive even higher dose compared to the relaxed position of the rectum when a balloon is not utilized. By using a rectal balloon, the dose exposure to the posterior rectal wall is decreased as opposed to an increased dose to the anterior rectal wall.^8^ This is illustrated in Fig. 2.

On the other hand, there is another clinically utilized technique in some clinics to spare the rectal dose via using materials such as hydrogel, hyaluronic acid gels, and collagen.^4^, ^5^ These techniques require an injection of a biodegradable spacer between the prostate and rectum, and they ultimately allow for approximately 1 cm perirectal space. Although these spacers will minimize the rectal dose, the biodegradable gel takes an average of 6 to 12 months to absorb once injected in the patients’ regions of interest. Moreover, it requires an invasive surgical procedure that demands attendance of a trained qualified physician to do the procedure guided by transrectal ultrasound to ensure the accurate localization and injection of the gel. Often times the procedure requires an additional MR‐based treatment planning to better visualize the gel and plan accordingly. Also, placement of the SpaceOAR^®^ hydrogel for patients with larger prostate glands tend to be more challenging to make sure it is injected properly and in a symmetrical fashion. The procedure takes anywhere from 10 to 30 min and sometimes under anesthesia. These lead to higher costs for the treatment procedure. It should also be noted that some patients have reported declines in bowel QOL or urinary QOL as well as gastrointestinal (GI) toxicity when the bioabsorbable gel is used. Uhl et al. in a study^9^ reported acute GI toxicity of Grade 0 or 1 of 88 percent, and Grade 2 of 12 percent in patients who had received the spacer gel prior to prostate radiation therapy. This toxicity was similar to another study conducted by Michalski et al.,^10^ who reported Grade 0 or 1 GI toxicity of 90 percent in patients, and Grade 2 of 10 percent for an IMRT dose of 79.2 Gy. There is also a risk of rectal ulcer associated with SpaceOAR^®^ hydrogel insertion as reported elsewhere.^11^ Other approaches that are currently being utilized are as ineffective and generally not accepted as standard of care.

This work aims to better understand the current trend in treatment of the tumors in pelvic cavity and present a novel organ repositioning device that was originally suggested by Parsai et al. and presented at AAPM annual conferences,^12^, ^13^ and later investigation was carried out to do the safety and feasibility studies in physically moving the rectum away from the path of a direct radiation during external beam and brachytherapy treatments using fresh cadaver. The result of that study will be presented in a follow‐up manuscript. The foundational methodology is to actuate a Nitinol shape memory wire that has been placed in an endorectal balloon, actively moving the rectum away from the path of radiation beam in a controlled manner. Such a device would not only work to mitigate morbidities to the rectum, but will also create the potential to deliver an ablative dose to tumor volume or to escalate the dose when needed.

In the presented methodology, a Nitinol actuator is incorporated in a rectal balloon immobilizer that has been clinically approved and used for prostate radiation therapy (RT) to reduce prostate motion. The Nitinol alloy offers multiple advantages such as biocompatibility and biomimetic actuation (via a reversible crystalline phase transformation).^14^, ^15^, ^16^, ^17^, ^18^ Moreover, Nitinol is widely accepted and used in the orthopedic and medical device community (e.g., cardiovascular stents)^19^, ^20^, ^21^, ^22^, ^23^, ^24^ in different forms and shapes thanks to the additive manufacturing.^25^

Nitinol alloy has two crystalline structures: austenite and martensite, which allows for its unique and reversible shape memory effect. In the rectal retractor, before the insertion, the Nitinol is soft and flexible (i.e., martensitic phase). The device will be inserted into the rectum so that the marking on the device is pointed toward the posterior rectal wall. Barium is then injected into the rectal retractor to determine the position of the device inside the pelvic cavity. Once localized, the balloon is inflated and the Nitinol will be actuated via joule heating. The heating is controlled by adjusting the current through a controller. When heated, the Nitinol element transforms to the austenitic phase, changes its shape, and as a result creates the desired motion. The retractor device pushes the posterior rectum wall, which will in turn move the anterior rectal wall as well away from the radiation field. Upon completion of the treatment, the actuator will trigger the positioner so it returns to its original shape (i.e., martensitic phase) so the device can be removed with patient's comfort. Due to the high fatigue resistance of the NiTi alloy, the core component can be used many times, which in turn reduces the cost of the procedure. Fatigue studies for such biomedical wires with various geometries are extensively studied elsewhere.^26^, ^27^, ^28^

The rectal retractor device also offers an open distal end to allow the integration of the marker with a barium delivery system for onboard imaging application which allows the device to serve a dual function of both providing a desirable geometry for a positioner device and facilitating the localization when imaging the area.

## 2 MATERIALS AND METHODS

The COMSOL Multiphysics software allowed for the simulation of the SMA wire's actuation and it's interfacing with the human body with accurate physiological and material properties. Heat transfer and the displacement of the rectal re‐positioner were simulated using the Joules Heating, Bio Heat Transfer, and Structural Dynamics modules.

The device was designed for easy insertion, based on anatomical constraint, and the NiTi shape memory alloy's features were exploited through cooling and heating the core alloy also known as shape setting for programming the memory effects.^14^, ^29^ Nitinol shape memory alloy core for rectal repositioning application required a custom shape in order to conform the geometry of a typical rectum. Shape setting (i.e., training of the NiTi core) was accomplished by constraining the Nitinol wire on a fixture to the desired geometry, which is here the actuated form, and applying a programmed heat treatment. Heat treatment includes complex thermo‐mechanical procedures that significantly affect functional and structural properties of the alloy.^14^ The dimensions as well as the shape setting parameters to set the shape and the properties of the core alloy were determined via multiple experiments in the lab to ensure the geometry of the Nitinol core is satisfactory considering the required force to move the rectum away from the path of radiation beam in posterior direction and an appropriate displacement is made. The time and temperature for NiTi shape setting were selected based on the literature^30^ as 500°C for 10 min in an Alumina fluidized bath.

Finally, the temperature of the actuator was precisely monitored for patients’ safety using the Micro‐Epsilon TIM camera and the prototype's actuation and displacement was tested with an experimental design that utilized an Agilent Power Supply E3631A, DS1104 R&D Controller Board, Micro‐Epsilon optoNCDT, Control Desk, and MATLAB programming Software.

In addition to the evaluation of the performance of the device, we have performed a preliminary cadaveric experiment to ensure that the suggested rectal retractor is able to displace the rectal tissue. A full and detailed cadaveric test will be published in a separate paper.

## 3 RESULTS AND DISCUSSION

### 3.A Simulation and experimental evaluation of the proposed device

**Figure 3:**
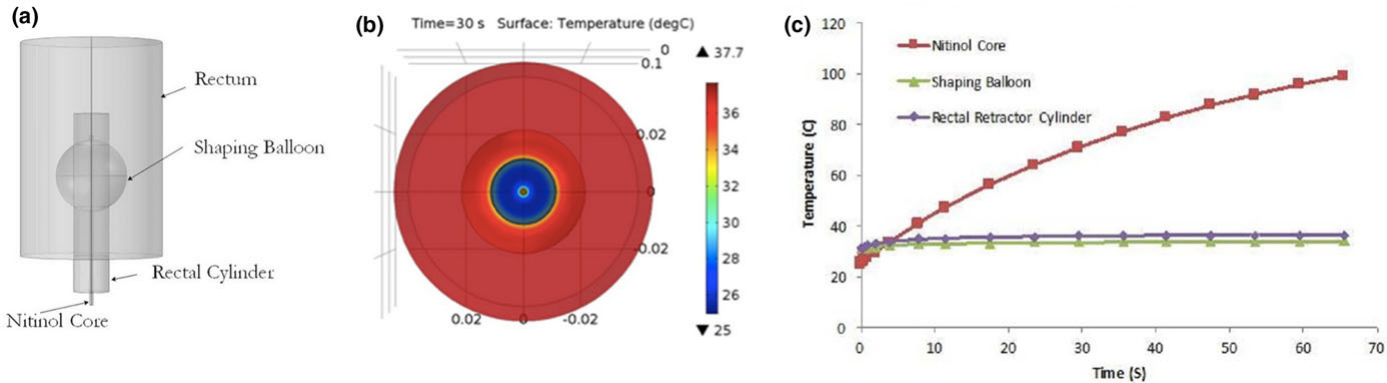
COMSOL Simulation: device design (a), temperature of cross‐sectional area of the device (b), and Temperature of the simulated device (c). [Correction added on 3 January 2019, after first online publication: the article has been corrected for Figure 3 after original publication.]

After the prototype was modeled and tested in COMSOL, the results were captured and are depicted in Fig. 3.

These values were then compared to the experimental data that were obtained in the Dynamic and Smart Systems Lab at University of Toledo School of engineering.

As seen in Fig. 3, one can obtain a better understanding about the feasibility and safety of the rectal repositioning device. Figure 3(a) reveals the rectal repositioning device that was modeled with its three cardinal components: Nitinol wire, flexible cylinder shaft, and the balloon. This prototype allows the device to first stabilize the rectum via inflating the balloon and then distance it away from the prostate when the Nitinol core is actuated using an external controller device.

**Figure 4:**
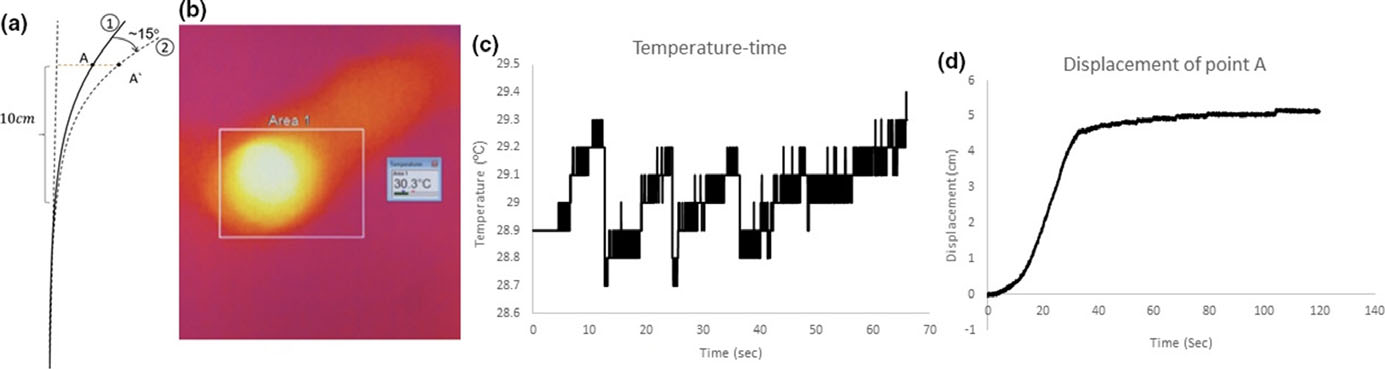
Prototype testing: schematic displacement of the actuator (a) thermal imaging (b), temperature of actuated device (c), and displacement profile (d).

Next, the heat dissipation and respective temperature profile of the device's components were studied; these experiments served to research whether the surface of the device that interfaced with the rectum would introduce hyperthermia and the associated heat shock response. In Fig. 3(b), the cross‐sectional area of the rectal balloon and Nitinol wire is visualized utilizing the COMSOL Multiphysics simulation package. The specific temperatures of the components were then assessed and shown in Fig. 3(c); the graph served to reveal the changes in temperature with the progression of time for the rectal balloon, the rectal cylinder, and the Nitinol wire. The temperature of the Nitinol wire reached 32.5°C. More importantly, the temperatures of the cylinder and balloon that interfaced with the human body reached to 34.0 and 36.2°C, respectively, upon being actuated for 60 s. It should be noted that the temperatures of the components would be less than these maximum values as the device would be operated for much less than 60 s. The simulation data were then compared to the data that were obtained from the rectal retractor's testing. Figure 4(a) shows the schematic displacement of the actuator. In Fig. 4(b), the region of the device where the electrical source is attached to the wire is shown as it occurs to see the highest heat concentration within the device.

Upon operating the device for 30 s, this region of the wire was found to have a temperature of 30.3°C, which is a negligible difference from the simulated 30.0°C. Furthermore, the temperature of the flexible cylinder (covering the Nitinol wire) after it was operated for 70 s was approximately 30.0°C in contrary to the simulated 32.5°C; however, for the clinical application of this device, less than 60 s of operating time is preferred before the patient is ready for imaging.

In the second series of prototype testing, the device's displacement between the rectum and prostate was studied. Specifically, the experiment was to obtain the qualitative relationship that would correlate the resistance of the Nitinol wire to the magnitude of the device's displacement. The results revealed that the maximum displacement is obtained around 30 s of operation with a 5 A current supply. This mathematical design will allow for safe and controlled actuation within the rectum. It is worth noting that the displacement test was done while there is no external load on the actuator. Another set of experiments were done to verify the actuator is able to apply up to 7N loads while actuating. In the case of existence of a 7N external load, the maximum displacement of the device was reported as 1 cm.

### 3.B Preliminary cadaverous test

**Figure 5:**
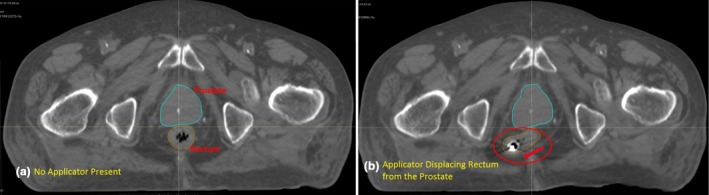
Computed tomography scan of the cadaver test of the rectal retractor, (a) before actuation (b) after actuation.

In order to ensure the feasibility of the rectal displacement, we have tested the proposed rectal retractor on a fresh cadaver. Figure 5 shows the CT scan image of the cadaver before and after activation of the rectal retractor in a preliminary study. As shown in the figure, the rectum has been displaced due to the activation of the rectal retractor and moved away from the prostate.

**Figure 6:**
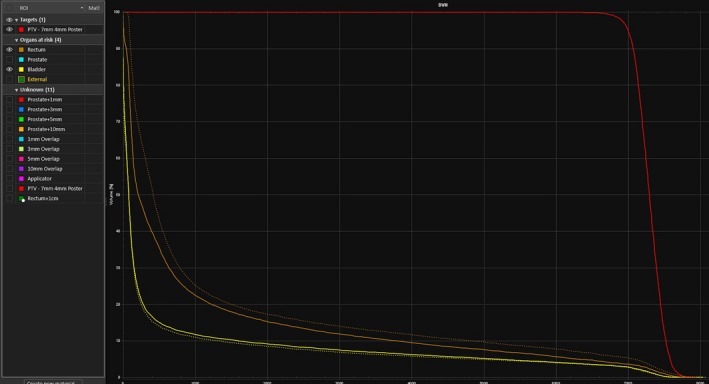
DVH diagram comparing without (dashed lines) and with (solid lines) the rectal applicator in place and actuated.

The retractor used for this study was an earlier version of the rectal retractor device and as shown in the CT scan, upon actuation moved the rectal tissue on one side of the coccyx bone, but it still moved the majority of the rectum away from the prostate. The newer prototypes have been designed for even more effective displacement of the rectum. A treatment plan generated using the cadaver CT scan for with and without the applicator in place indicates a significant sparing of rectal tissue by comparing the DVH diagram shown in Fig. 6. In addition, it can be concluded from this preliminary study that the prostate gland is not affected due to displacement of the rectum. In fact, the perirectal fat between the prostate and rectum has a higher elasticity than rectal tissue seems to have stretched safely allowing a distance between the rectum and prostate without causing significant damage to the whole rectal tissue. A more detailed and comprehensive report on the cadaveric test of the modified device will be published in the near future.

## 4 CONCLUSIONS

With most pelvic tumors benefiting from radiation, especially in younger patients with longer life expectancy, the success of treatment and the absence of late term radiation induced complications is a direct function of how the surrounding and normal tissue are spared during the course of radiotherapy. Ample literature in recent years indicates that radiation‐induced chronic morbidities in critical structures located in vicinity of or in the path of radiation fields are common and RT‐induced second malignancies will likely increase^31^ if care is not taken to reduce dose to those structures. Moving these structures away from the region of radiotherapy provides an effective means for reducing the risks of morbidities to these structures and increasing the therapeutic dose to the target volume. For most cancers of the pelvis, the rectum is considered a key dose‐limiting organ where increased rectal dose can lead to acute proctitis and potential serious late toxicities, including chronic irritation, bleeding, or ulceration. For instance, in treatment of prostate cancer using radiotherapy, we currently have no easy or effective solution to place a distance between rectum and the field of radiation. This is true for conventional EBRT, IMRT, and HDR brachytherapy.

The authors suggest a retracting device whereby the rectal wall can be shifted away from the source of radiation, or from vicinity of the radiation field. This will result in lower dose to this dose‐limiting structure, hence creating potentials to deliver ablative dose to tumor volume, or escalate dose when needed. Moving the rectum away even a short distance from the plane of radiation could dramatically relax the prescription dose limitation, particularly in HDR brachytherapy treatments and also in SBRT treatments of the pelvic tumors.

The COMSOL simulation and the prototype testing data are strongly suggestive of the device not inducing patients risks such as hyperthermia, which allows for patient safety and comfort during operation. Specifically, the temperature of the prototype device at the hottest portion will not surpass 30.0°C in experimentation.

The displacement testing allowed for a correlation between the displacement of the rectum and the Nitinol wire's resistance; a design equation details when the rectum has been displaced 1.0 cm so that the current supply may be terminated. Data suggest that the device displaces the rectum 1.0 cm after it has been activated for less than 30 s, which is appropriate during prostate cancer radiotherapy for an efficient dose sparing to the rectum. The prototype device is able to apply as high as 7N loads while creating a displacement of 1 cm. For any other cases with lower level of resistance (i.e., resistance of the rectal tissue), the required displacement (i.e., 1 cm displacement of the rectal tissue) will occur before 30 s and can be controlled using the controller unit.

## CONFLICT OF INTERESTS

None to report.
